# Supplementary material for: Cytosolic ROS production by NADPH oxidase 2 regulates muscle glucose uptake during exercise
Source: Nat Commun. 2019 Oct 11;10:4623. doi: 10.1038/s41467-019-12523-9 (PMC6789013; doi:10.1038/s41467-019-12523-9)
Supplement: Supplementary file 1 — Supplementary Information [file 41467_2019_12523_MOESM1_ESM.pdf]

Cytosolic ROS production by NADPH oxidase 2 regulates muscle glucose uptake during exercise

Carlos Henríquez-Olguin *et al.*

Supplementary Information

Supplementary Table 1. Antibodies used in the study.

Antibody	Supplier	Catalog #	Dilution
p-AMPK Thr172	Cell Signaling Technology	#2535S	1:1000
p-p38 MAPK Thr180/Tyr182	Cell Signaling Technology	#9211	1:1000
Hexokinase II	Cell Signaling Technology	#2867	1:1000
GLUT4	Invitrogen	#PA5-23052	1:1000
p-ACC2 Ser 212	Millipore	#03-303	1:1000
Rac1	BD Biosciences	#610650	1:1000
NOX2	Abcam	#Ab129068	1:1000
Catalase	Santa Cruz Biotechnology	sc-271803	1:750
MnSOD	Millipore	#06-984	1:1000
TRX2	Santa Cruz Biotechnology	#sc-50336	1:1000
Actin	Cell Signaling Technology	#4973	1:3000
Total p38 MAPK	Cell Signaling Technology	#9212	1:1000
Alpha2 AMPK	D. Grahame Hardie, University of Dundee		1:1000
p-Erk1/2-Thr202/Tyr204	Cell Signaling Technology	#9101	1:1000
Total ERK 1/2	Cell Signaling Technology	#9102	1:1000
p-TBC1D1 Ser231	Millipore	#07-2268	1:1000
OXPHOS cocktail	Abcam	#ab110413	1:5000
Goat-anti-mouse IgG2b Alexa 647 conjugated	Life Technologies	A-21242	1:100
Goat-anti-mouse IgM, Alexa 555 conjugated	Life Technologies	A-21426	1:500
Goat-anti-mouse IgG1 Alexa 488 conjugated	Life Technologies	A-21121	1:400
Myosin Heavy Chain Type I	DSHB, University of Iowa	BA-D5	1:100
Myosin Heavy Chain Type IIA	DSHB, University of Iowa	SC-71	1:100
Myosin Heavy Chain Type IIB	DSHB, University of Iowa	BF-F3	1:100
PECAM1	Santa Cruz Biotechnology	M-20	1:100
Myc	Cell Signaling Technology	#2278	1:400
Cu-Zn-SOD			
Alexa 568 secondary antibody	Life Technologies	A10042	1:400 diluted blocking buffer containing 0.04% saponin

Supplementary Table 2. Human subjects’ characteristics.

	Mean	SD
Age (y)	29.0	3.56
Weight (kg)	76.8	7.03
Height (cm)	1.76	0.07
BMI (m/kg^2)	24.9	1.45
Peak Power Output (W)	300	67.17
Relative Peak Power Output (W/kg)	3.9	0.51

Supplementary Figures

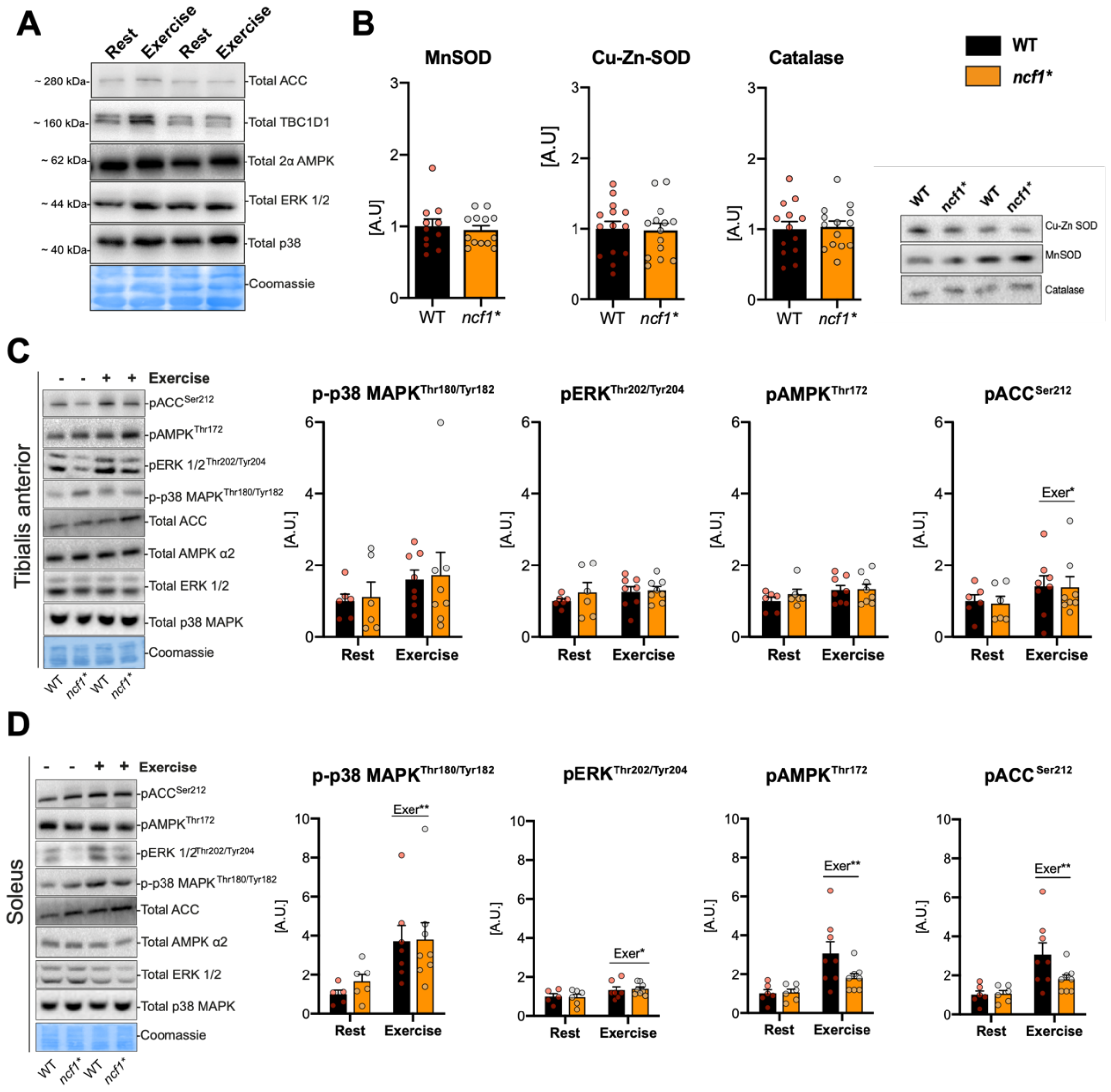

Supplementary Figure 1. Similar abundance of antioxidant enzymes in *ncf1 muscles. a** Total proteins levels of p38 MAPK, ERK 1/2, alpha2 AMPK, ACC, TBC1D1 and Coomassie staining as loading control in human muscle lysates (n=3). **b** Antioxidant proteins from tibialis anterior muscle lysates from both *ncf1** and WT mice (n=13-14). **c, d** Exercise-induced cellular signaling in soleus and tibialis anterior muscles. In **b,** a t-test was performed for statistical analysis. For **c, d** two-way ANOVA was performed to test for effects of exercise (Exer) genotype (Geno), and interaction (Int), followed by Tukey's

post hoc test with correction for multiple comparisons. *, ** denotes $p < 0.05$ and $p < 0.01$, respectively for main effects. Individual values and mean ± SEM are shown.

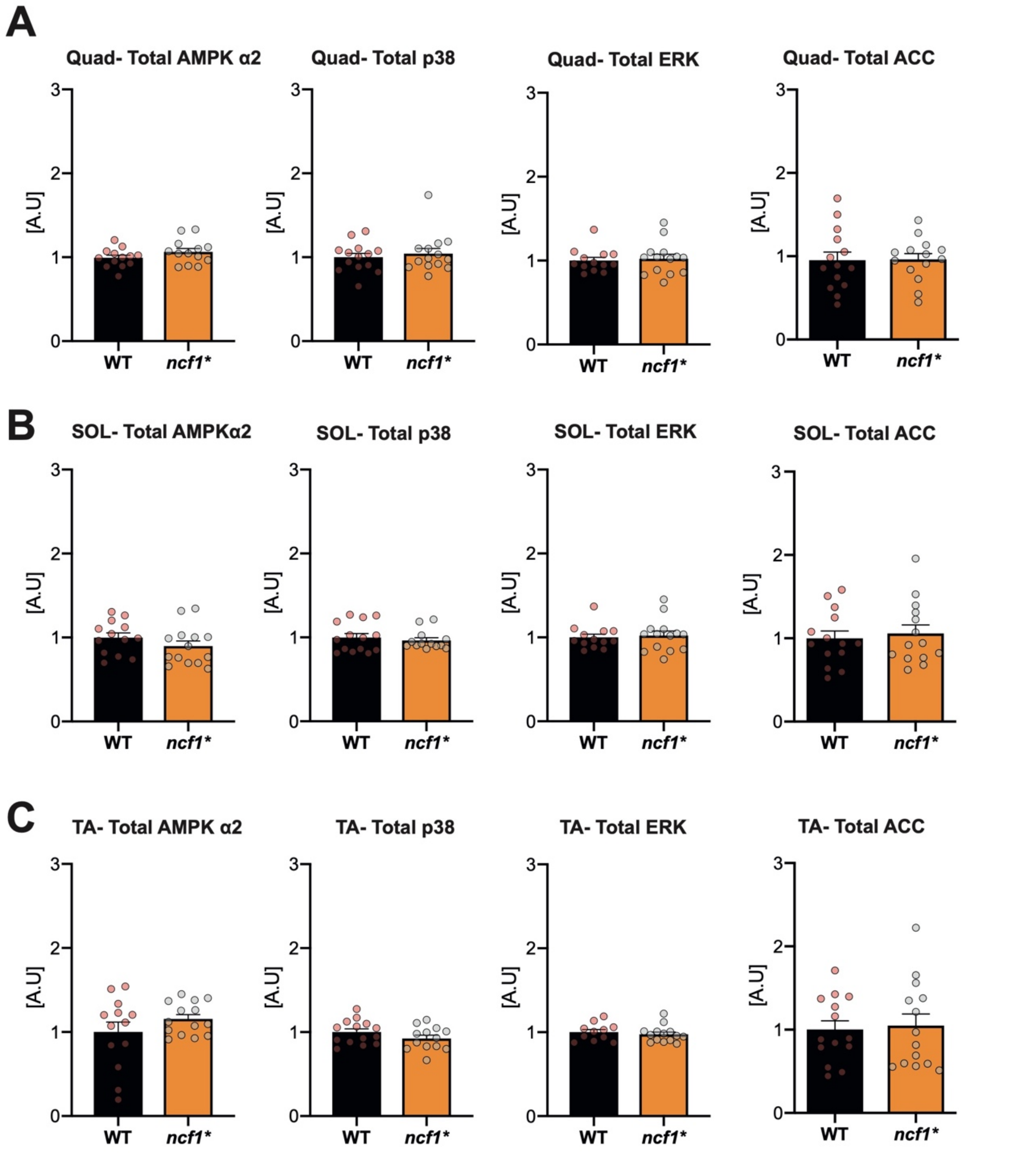

Supplementary Figure 2. Total protein levels of exercise-responsive kinases are similar in WT and *ncf1** muscles. Protein levels of p38 MAPK, ERK 1/2, alpha2 AMPK, and ACC in **a** quadriceps **b** soleus (SOL) and **c** tibialis anterior (TA) from WT and *ncf1** mice (n=14 per group). For **a**, **b,** and **c** t-test were performed for statistical analysis. Individual values and mean ± SEM are shown.

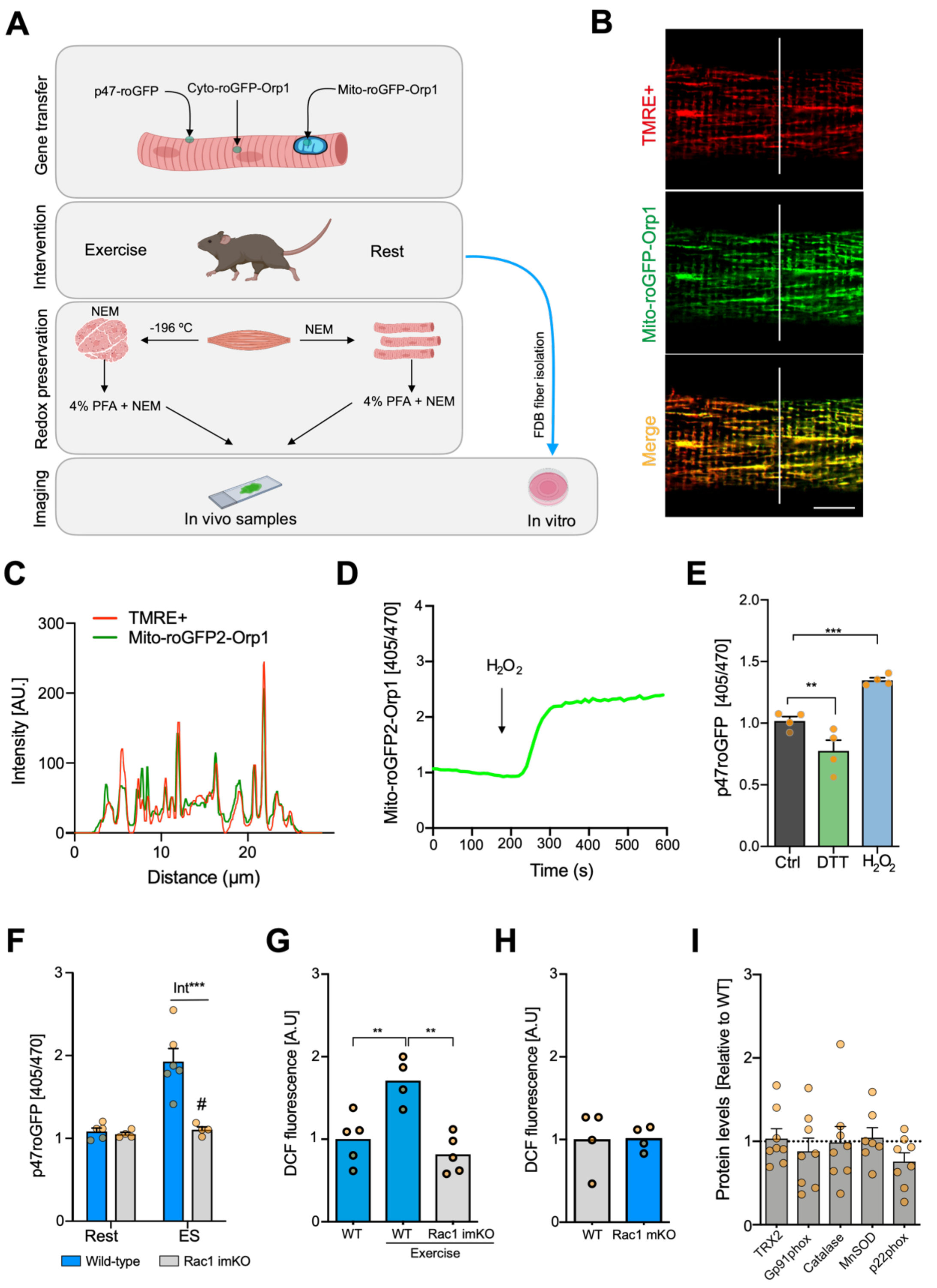

Supplementary Figure 3. Genetically-encoded biosensors are able to measure subcellular redox changes in muscle fibers. **a** Graphical description of the work flow for genetically encoded redox biosensors (the procedures are described in the methods section) **b** Representative image and **c** line-profile of the Mito-roGFP2-Orp1 and TMRE^{+}

fluorescence in live flexor digitorum brevis (FDB) fibers (n=3). **d** Mito-roGFP2-Orp1 oxidation by H_2O_2 (0.5 mM) stimulation in live FDB fibers (n=3). **e** p47roGFP oxidation/reduction in response to H_2O_2 (0.5 mM) and DTT (0.5 mM) (n=4). **f** p47roGFP oxidation induced by electrical stimulation in FDB Rac1 imKO fibers (*n*=5). **g** DCFH oxidation after 20 min of exercise in WT and Rac1 imKO Tibialis Anterior muscle (*n*=5-4). **h** DCFH oxidation under resting conditions in WT and Rac1 imKO tibialis anterior muscle. **i** Redox-regulated proteins in FDB fibers are similar between WT and Rac1 deficient muscles (*n*=4-5). One- way (for E, G, I) and two-way ANOVA (for **f**) were performed to test for effects of exercise (Exer), genotype (Geno), and interaction (Int), followed by Tukey's *post hoc* test with correction for multiple comparisons. Individual values and mean ± SEM are shown. # denotes $p< 0.05$ compared to the WT group. **, *** denotes $p< 0.01$ and $p< 0.001$ respectively for main effect. For **b** scale bar= 10 um.

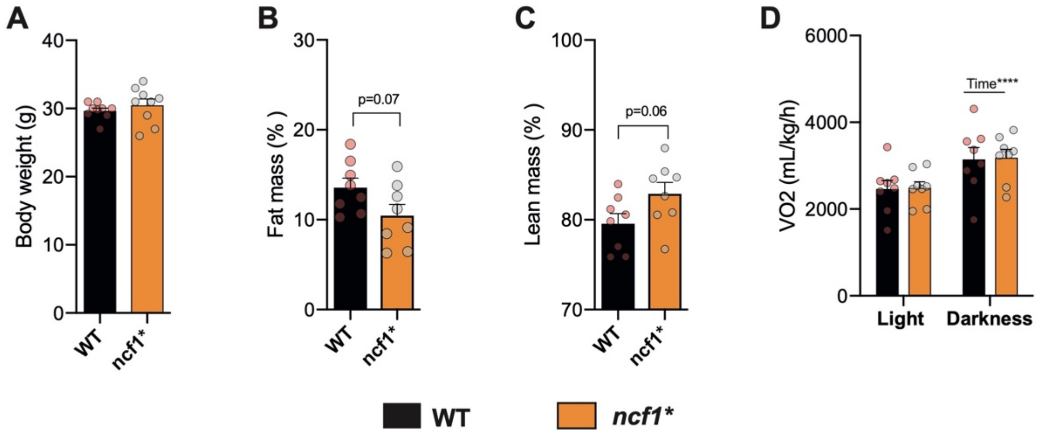

Supplementary Figure 4. Body weight and composition of *ncf1** mice. A) Similar body weight despite tendency to B) lower body fat, C) higher lean mass and D) equal oxygen consumption in *ncf1** compared to WT mice. Unpaired t-test (F) and two-way ANOVA were performed to test for effects of exercise (Exer) genotype (Geno), and interaction (Int), followed by Tukey's *post hoc* test to compare groups. **** denotes $p< 0.0001$ respectively for main effects. Individual values and mean ± SEM are shown.

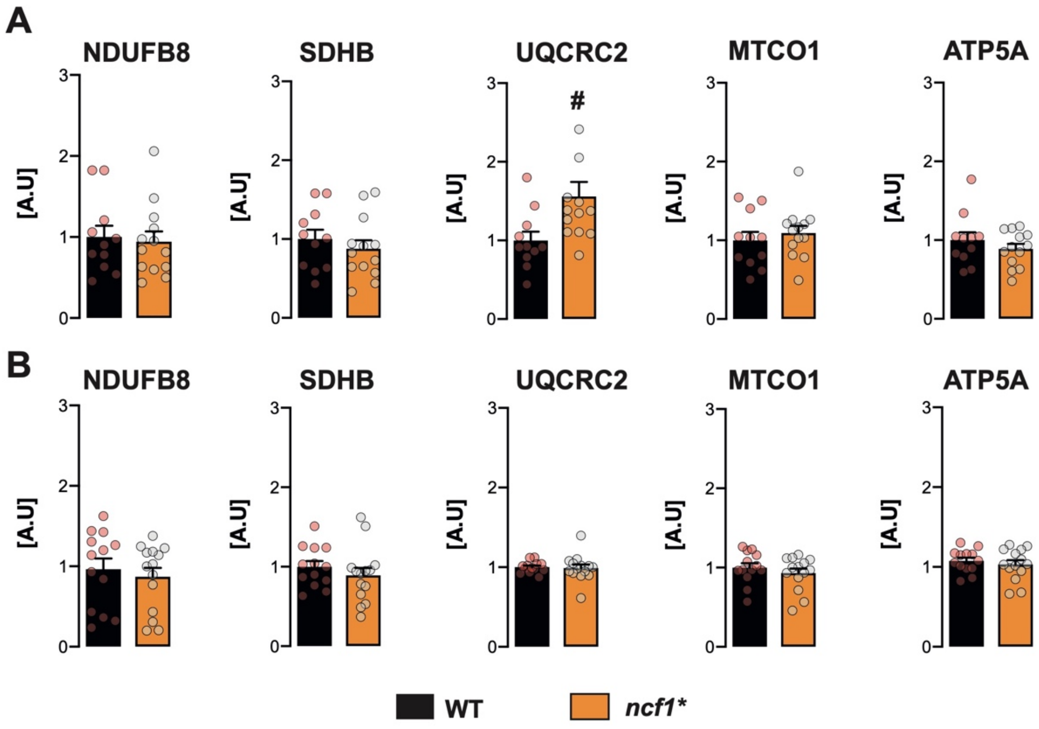

Supplementary Figure 5. Similar content of mitochondrial oxidative phosphorylation complex proteins in *ncf1** compared to WT muscles. Quantification graphs of total protein showed figure 3E-F. A) quadriceps (n=12-13) and B) soleus muscle (n=13-14). A t-test was performed for statistical analysis. Individual values and mean ±SEM are shown.

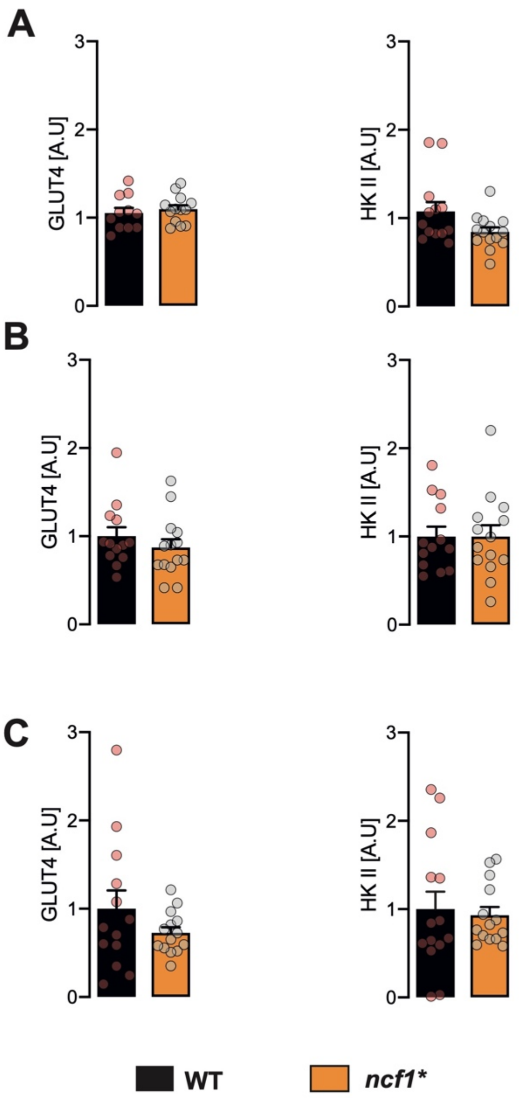

Supplementary Figure 6. Similar abundance of endogenous glucose handling-related proteins in *ncf1** and WT muscles. Quantification graphs of total protein showed in figure 3B-D. A) quadriceps B) soleus, and C) tibialis anterior (TA) muscles. A t-test was performed for statistical analysis. Individual values and mean ±SEM are shown.
