# Supplementary material for: Cytosolic ROS production by NADPH oxidase 2 regulates muscle glucose uptake during exercise
Source: Nat Commun. 2019 Oct 11;10:4623. doi: 10.1038/s41467-019-12523-9 (PMC6789013; doi:10.1038/s41467-019-12523-9)
Supplement: Supplementary file 2 — Reporting Summary [file 41467_2019_12523_MOESM2_ESM.pdf]

nature research

Corresponding author(s): Thomas E Jensen

Last updated by author(s): 30-04-2019

Reporting Summary

Nature Research wishes to improve the reproducibility of the work that we publish. This form provides structure for consistency and transparency in reporting. For further information on Nature Research policies, see Authors & Referees and the Editorial Policy Checklist.

Statistics

For all statistical analyses, confirm that the following items are present in the figure legend, table legend, main text, or Methods section.

n/a	Confirmed	
☐	☒	The exact sample size (*n*) for each experimental group/condition, given as a discrete number and unit of measurement
☐	☒	A statement on whether measurements were taken from distinct samples or whether the same sample was measured repeatedly
☐	☒	The statistical test(s) used AND whether they are one- or two-sided<br>*Only common tests should be described solely by name; describe more complex techniques in the Methods section.*
☒	☐	A description of all covariates tested
☐	☒	A description of any assumptions or corrections, such as tests of normality and adjustment for multiple comparisons
☐	☒	A full description of the statistical parameters including central tendency (e.g. means) or other basic estimates (e.g. regression coefficient) AND variation (e.g. standard deviation) or associated estimates of uncertainty (e.g. confidence intervals)
☒	☐	For null hypothesis testing, the test statistic (e.g. *F*, *t*, *r*) with confidence intervals, effect sizes, degrees of freedom and *P* value noted<br>*Give P values as exact values whenever suitable.*
☒	☐	For Bayesian analysis, information on the choice of priors and Markov chain Monte Carlo settings
☒	☐	For hierarchical and complex designs, identification of the appropriate level for tests and full reporting of outcomes
☒	☐	Estimates of effect sizes (e.g. Cohen's *d*, Pearson's *r*), indicating how they were calculated

Our web collection on statistics for biologists contains articles on many of the points above.

Software and code

Policy information about availability of computer code

Data collection	No software was used
Data analysis	Statistical analysis were performed using GraphPad Prism 7 sofware (GraphPad, Inc), FIJI.

For manuscripts utilizing custom algorithms or software that are central to the research but not yet described in published literature, software must be made available to editors/reviewers. We strongly encourage code deposition in a community repository (e.g. GitHub). See the Nature Research guidelines for submitting code & software for further information.

Data

Policy information about availability of data

All manuscripts must include a data availability statement. This statement should provide the following information, where applicable:
- Accession codes, unique identifiers, or web links for publicly available datasets
- A list of figures that have associated raw data
- A description of any restrictions on data availability

Provide your data availability statement here.

Field-specific reporting

Please select the one below that is the best fit for your research. If you are not sure, read the appropriate sections before making your selection.

☒ Life sciences ☐ Behavioural & social sciences ☐ Ecological, evolutionary & environmental sciences

For a reference copy of the document with all sections, see nature.com/documents/nr-reporting-summary-flat.pdf

Life sciences study design

All studies must disclose on these points even when the disclosure is negative.

Sample size	No statistical method was used to predetermine animal's sample size. The sample size was determined based on experience with the used experimental models/setups. The precise number of animals used are given in the figure legend.
Data exclusions	No samples were excluded from analyses.
Replication	No attempts at experimental replication failed.
Randomization	Mice were randomized in each study.
Blinding	Maximal running test, microscopy imaging and western blot analysis were performed blinded.

Reporting for specific materials, systems and methods

We require information from authors about some types of materials, experimental systems and methods used in many studies. Here, indicate whether each material, system or method listed is relevant to your study. If you are not sure if a list item applies to your research, read the appropriate section before selecting a response.

Materials & experimental systems

n/a	Involved in the study	
☐	☒	Antibodies
☒	☐	Eukaryotic cell lines
☒	☐	Palaeontology
☐	☒	Animals and other organisms
☐	☒	Human research participants
☒	☐	Clinical data

Methods

n/a	Involved in the study	
☒	☐	ChIP-seq
☒	☐	Flow cytometry
☒	☐	MRI-based neuroimaging

Antibodies

Antibodies used

p-AMPK Thr172 (Cell Signaling Technology (CST), #2535S), Dilution 1:1000
p-p38 MAPK Thr180/Tyr182 (CST, #9211), Hexokinase II (CST, #2867) Dilution 1:1000
GLUT4 (PA5-23052, Invitrogen) Dilution 1:1000
p-ACC2 Ser 212 (Millipore, 03-303) Dilution 1:1000
ACC protein was detected using horseradish peroxidase-conjugated streptavidin from Dako (P0397), Dilution 1:3000
Rac1 (BD Biosciences, #610650) Dilution 1:1000
NOX2 (Abcam, #Ab129068) Dilution 1:1000
Catalase (SCBT, sc-271803) Dilution 1:750
MnSOD (Millipore, 06-984) Dilution 1:1000
TRX2 (SCBT, sc-50336) Dilution 1:1000
Actin (CST, #4973) Dilution 1:3000
Total p38 MAPK (CST, #9212) Dilution 1:1000
p-P38MAPK- Thr180/Tyr182 (CST, #9211)
Alpha2 AMPK (a gift from D. Grahame Hardie, University of Dundee)
pErk1/2-Thr202/Tyr204 (CST, #9101) Dilution 1:1000
Total ERK 1/2 (CST, #9102) Dilution 1:1000
TBC1D1 ser231 (Millipore #07-2268) Dilution 1:1000
OXPHOS cocktail (Abcam, #ab110413) Dilution 1:5000
Goat-anti-mouse IgG2b Alexa 647 conjugated (Life Technologies, Carlsbad, USA)
Goat-anti-mouse IgG1 Alexa 488 conjugated (Life Technologies, Carlsbad, USA)
Goat-anti-mouse IgM, Alexa 555 conjugated (Life Technologies, Carlsbad, USA)
BA-D5 (DSHB, University of Iowa) Dilution 1:100
SC-71(DSHB, University of Iowa) Dilution 1:100
BF-F3 (DSHB, University of Iowa) Dilution 1:100
anti-PECAM1 antibody (SCBT, M-20) diluted 1:100

Validation

All the antibodies used in this study were validated by providers

Animals and other organisms

Policy information about studies involving animals; ARRIVE guidelines recommended for reporting animal research

Laboratory animals	Inducible muscle-specific male Rac1 mice (imKO) and littermate control mice were generated by crossbreeding Rac1 fl/fl mice. Male B10.Q wild-type (WT) and B10.Q. p47phox mutated (ncf1*) mice.
Wild animals	No wild animals were used in this study.
Field-collected samples	No field-collected samples were used in this study.
Ethics oversight	All experiments were approved by the Danish Animal Experimental Inspectorate (2015–15–0201–00477).

Note that full information on the approval of the study protocol must also be provided in the manuscript.

Human research participants

Policy information about studies involving human research participants

Population characteristics	Human participants include healthy young male subjects (Age 29 +/- 3.56y BMI 24.8 +/- 1.76 kg/m2)
Recruitment	Research subjects were recruited by advertising the study at our Department
Ethics oversight	Regional Ethics Committee for Copenhagen (H-16040740)

Note that full information on the approval of the study protocol must also be provided in the manuscript.
